# DC-SIGN and CD150 Have Distinct Roles in Transmission of Measles Virus from Dendritic Cells to T-Lymphocytes

**DOI:** 10.1371/journal.ppat.1000049

**Published:** 2008-04-18

**Authors:** Lot de Witte, Rory D. de Vries, Michiel van der Vlist, Selma Yüksel, Manja Litjens, Rik L. de Swart, Teunis B. H. Geijtenbeek

**Affiliations:** 1 Department of Molecular Cell Biology and Immunology, VU University Medical Center, Amsterdam, The Netherlands; 2 Department of Virology, Erasmus MC, University Medical Center, Rotterdam, The Netherlands; University of California Irvine, United States of America

## Abstract

Measles virus (MV) is among the most infectious viruses that affect humans and is transmitted via the respiratory route. In macaques, MV primarily infects lymphocytes and dendritic cells (DCs). Little is known about the initial target cell for MV infection. Since DCs bridge the peripheral mucosal tissues with lymphoid tissues, we hypothesize that DCs are the initial target cells that capture MV in the respiratory tract and transport the virus to the lymphoid tissues where MV is transmitted to lymphocytes. Recently, we have demonstrated that the C-type lectin DC-SIGN interacts with MV and enhances infection of DCs *in cis*. Using immunofluorescence microscopy, we demonstrate that DC-SIGN^+^ DCs are abundantly present just below the epithelia of the respiratory tract. DC-SIGN^+^ DCs efficiently present MV-derived antigens to CD4^+^ T-lymphocytes after antigen uptake via either CD150 or DC-SIGN *in vitro*. However, DC-SIGN^+^ DCs also mediate transmission of MV to CD4^+^ and CD8^+^ T-lymphocytes. We distinguished two different transmission routes that were either dependent or independent on direct DC infection. DC-SIGN and CD150 are both involved in direct DC infection and subsequent transmission of *de novo* synthesized virus. However, DC-SIGN, but not CD150, mediates *trans-*infection of MV to T-lymphocytes independent of DC infection. Together these data suggest a prominent role for DCs during the initiation, dissemination, and clearance of MV infection.

## Author Summary

Despite the availability of an effective vaccine, measles virus (MV) is still a major cause of childhood morbidity and mortality in developing countries. Almost all non-vaccinated children catch the highly contagious virus during an MV outbreak. This suggests an efficient route for primary infection. However, the main target cells for MV replication, CD150^+^ lymphocytes, are barely present in the respiratory tract where MV enters the body. Here we demonstrate an alternative route of MV transmission: dendritic cells that are abundantly present in the sub-epithelial tissues of the respiratory tract may capture MV through binding to either CD150 or DC-SIGN. Although some virus particles are processed for antigen presentation, others escape from degradation. After virus capture, DCs migrate to the lymphoid tissues where they encounter CD150^+^ lymphocytes and transmit the virus, after which viral replication is started. Our data provide new insights into the transmission of measles virus, and suggest a dual role for DCs in the pathogenesis of measles.

## Introduction

Measles is a systemic disease, caused by measles virus (MV) infection of respiratory and lymphoid tissues. MV is a member of the *Paramyxoviridae* family, genus *Morbillivirus*. The virus is highly contagious and is spread via the respiratory route [1]. Although the course and symptoms of measles are well characterized, little is known about the cellular events underlying the disease. The target cells for MV at the site of transmission and during the systemic phase of the disease are still under debate [1]–[4]. Moreover, the interaction of MV with the immune system, paradoxically resulting in induction of strong MV-specific immunity, but also immunosuppression, has not been fully clarified.

It was previously thought that MV initially infects epithelial cells of the respiratory tract, and is disseminated during viraemia by infected monocytes [3],[5]. However, these cells only express CD46, the receptor for attenuated MV strains, but do not express CD150 (SLAM), the primary receptor for wild-type MV [6],[7]. CD150 is mainly expressed on subsets of lymphocytes, thymocytes, macrophages and mature dendritic cells (DCs) [7]. Moreover, we have recently shown that lymphocytes, but not monocytes, are the predominant cells infected *in vivo* during measles in macaques [2]. Moreover, lymphocytes are not in large numbers present at respiratory epithelial surfaces compared to lymphoid tissues and therefore we hypothesize that other cells are the target for MV at sites of entry.

DCs are professional antigen presenting cells (APCs) that have a sentinel function in the immune system; DCs capture antigens in the periphery and, upon activation, migrate to the lymphoid tissues to present the antigens to T-lymphocytes, resulting in a pathogen-specific immune response [8]. We hypothesize that DCs mediate transmission of MV: DCs capture MV in the respiratory tract, but instead of degradation the virus is protected and transported into the lymphocyte-rich area in the lymphoid tissues, where it is efficiently transmitted to CD150^+^ lymphocytes. A similar role for DCs has been described for HIV-1, where DCs capture HIV-1 via the C-type lectin dendritic cell-specific ICAM-3 grabbing non-integrin (DC-SIGN) and mediate transmission of HIV-1 to T-lymphocytes by *de novo* production of virus or transferring the virus particles directly to the T-lymphocytes (*trans-*infection) [9],[10]. We have previously shown that DC-SIGN also mediates binding of MV to DCs, which enhances DC infection through CD150 *in cis* [11]. Moreover, in infected macaques MV-infected DCs have been observed in conjunction with infected T-lymphocytes, suggesting transmission of virus between both cell types [2].

Here we set out to investigate the role of DC-SIGN and CD150 in both antigen presentation and MV transmission by DCs. MV capture by DCs leads not only to antigen presentation but also to efficient transmission to T-lymphocytes. Both the tissue distribution and functional studies demonstrate that CD150 and DC-SIGN have distinct functions in MV transmission by DCs. The identification of their function in antigen presentation and MV transmission will lead to a better understanding of MV pathogenesis.

## Results

### DC-SIGN^+^ dendritic cells are present in the respiratory tract and closely interact with CD150^+^ cells in lymphoid tissues

MV enters the body in the respiratory tract; however the initial target cells at the site of entry remain unknown. DC-SIGN and CD150 are the major receptors for wild-type MV strains, of which only CD150 can function as entry receptor. DC-SIGN is abundantly expressed by DCs in peripheral tissues, such as the dermis, foreskin, gut and cervix and on DCs and specialized macrophages in the lymphoid tissues [9], [12]–[14]. However, little is known about the expression of DC-SIGN and CD150 in the respiratory tract. Therefore we investigated the presence of DC-SIGN^+^ and CD150^+^ cells in the different respiratory tissues by immunofluorescence microscopy.

**Figure 1:**
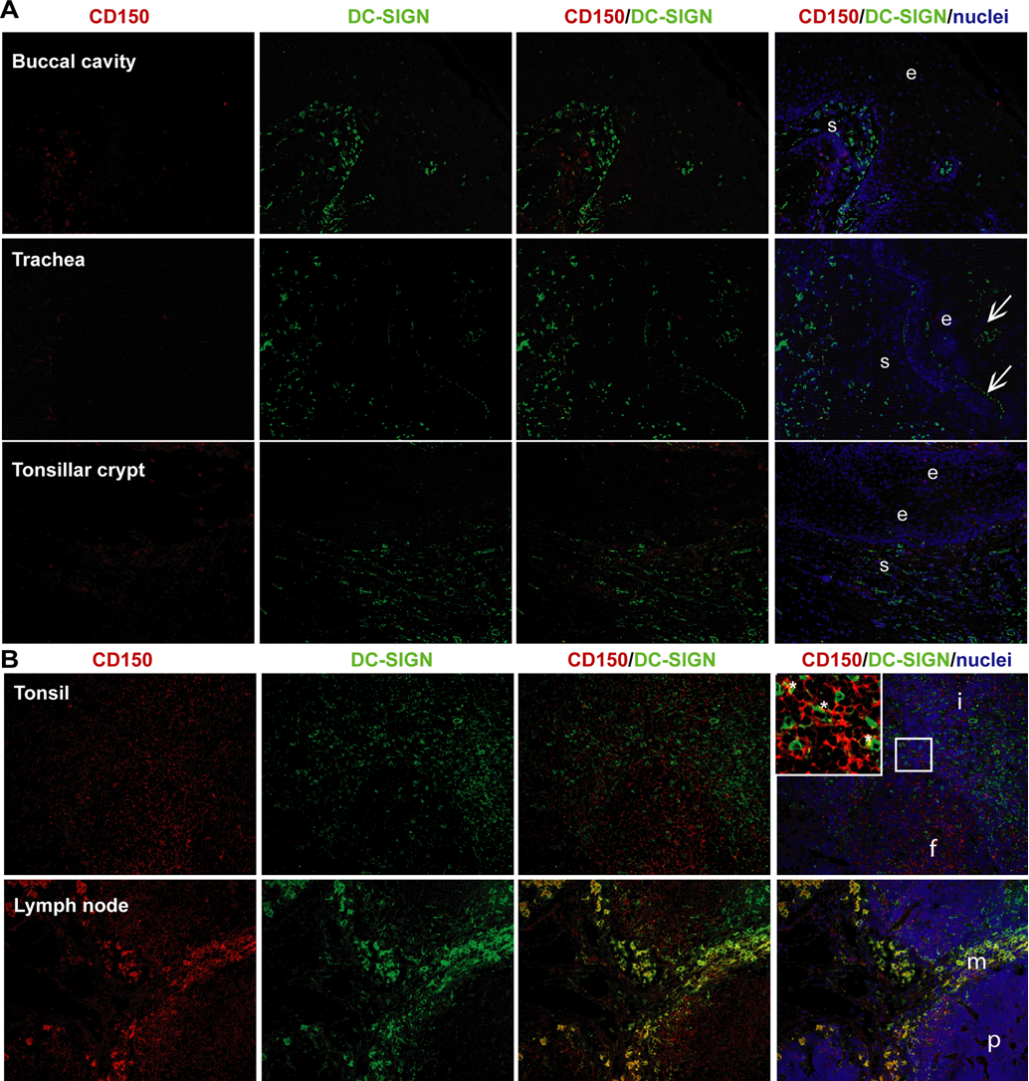
DC-SIGN^+^-dendritic cells are present in the sub-epithelial tissues of the respiratory tract. (A,B) Cryosections of different tissues from healthy donors were stained for the expression of DC-SIGN (green) and CD150 (red) using specific antibodies, and for the nuclei using Hoechst (blue). The sections were analyzed by fluorescence microscopy. (A,B) Representative photos with a magnification of 100× are depicted (e = epithelium; s = sub-epithelial tissue, i = inter-follicular, f = follicles, m = medullary sinus, p = paracortex, arrow = autofluorescence, * = co-localization).

DC-SIGN was abundantly present in buccal, pharyngeal, tonsillar, tracheal and bronchial sub-epithelial tissues (Figure 1A and S1, Table 1). Similar to previous reports [15], scattered DC-SIGN^+^ DCs were also observed in the lungs, mainly in the interstitium of the alveoli (Table 1).

**Table 1:** DC-SIGN^+^-dendritic cells are present in the sub-epithelial tissues of the respiratory tract.

	Tissues		CD150#	DC-SIGN	Co-localization	n
Upper respiratory tract	Buccal cavity	Epithelium	0	0	0	1
		Sub-epithelium	+	+++	0/+	
	Pharynx	Epithelium	+	0	0	1
		Sub-epithelium	+	+++	0	
	Tonsillar crypt	Epithelium	0/+	0	0	2
		Sub-epithelium	+	+++	0	
Lower respiratory tract	Trachea	Epithelium	0	0	0	2
		Sub-epithelium	0/+	++	0	
	Bronchus	Epithelium	0	0	0	2
		Sub-epithelium	0	++	0	
	Lung	Interstitium	0/+	0/+	0	2
Lymphoid tissue	Tonsil	Folicle	+++	+++	++	3
		Inter-folicular	+++	+++	++	
	Lymph node	B-cell area	+++	+	+	1
		T-cell area	+++	++	++	
		Medullary sinus	+++	+++	+++	

# The number of DC-SIGN^+^- or CD150^+^- cells was determined by counting the number of positive cells, divided by the total number of cells, based on the nuclei staining. The double-positive cells were divided by the total number of stained cells, to determine the co-localization. 0 = 0–1%, + = 1–5%, ++ = 5–25%, +++ = 25–100% of the cells, n = number of donors analyzed per tissue.

Monocyte-derived DCs (moDCs) in culture express CD150, and expression levels are increased upon maturation [16],[17]. *In situ,* CD150 expression was detected on cells in the sub-epithelial tissues of the upper respiratory tract, but we observed very little expression in the lower respiratory tract (Figure 1A and S1, Table 1). Although some CD150^+^ cells were present in the epithelia of the tonsillar crypts and the pharynx, the expression of CD150 in these tissues was low compared to that in lymphoid tissues (Figure 1A and S1, Table 1). These cells might represent macrophages or lymphocytes that can be targets for MV infection and their infection might explain why infection is observed in epithelial tissues in vivo [2]. We also observed some autofluorescence in the epithelia of the tracheal, bronchial and tonsillar epithelium, probably caused by mucus (Figure 1A and S1). In the upper respiratory tract, we rarely detected co-localization of CD150 and DC-SIGN, suggesting that DC-SIGN^+^ DCs, abundantly present just below the epithelia in the respiratory tract, express no or low levels of CD150 (Figure 1A and S1, Table 1).

During viraemia, MV-infected cells enter the lymphoid tissues. Here, DC-SIGN^+^ DCs might facilitate infection of lymphocytes, similar to HIV-1 [9]. We therefore investigated the expression of DC-SIGN and CD150 in lymph node and tonsil. As previously described [13],[15], DC-SIGN^+^ cells were mainly located around the medullary sinuses and in the paracortex (T-cell areas) of the lymph node as well as in the inter-follicular tissue (T-cell area) of the tonsil (Figure 1B, Table 1). However, DC-SIGN^+^ cells were also found in the B cell follicles of the tonsil. Notably, DC-SIGN^+^ cells were located in close contact with the CD150^+^ cells (Figure 1B). Strikingly, strong co-localisation of DC-SIGN and CD150 was observed in the medullary sinuses (Figure 1B, Table 1). Together these data suggest that DC-SIGN^+^ cells are not only important in the initial phase of MV infection, but might also be involved in MV infection in the lymphoid tissues during the systemic phase of the infection.

### Dendritic cells mediate antigen presentation of measles virus to CD4^+^ T-lymphocytes

**Figure 2:**
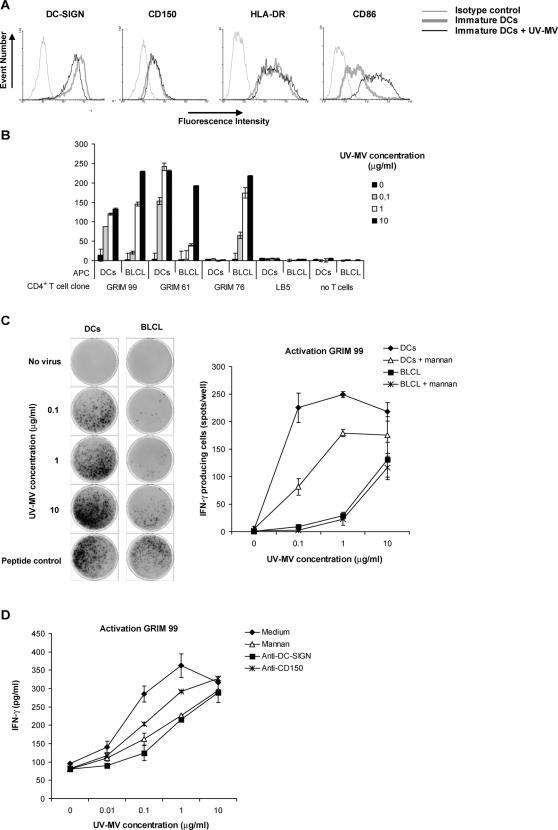
Dendritic cells mediate antigen presentation of measles virus to CD4^+^ T-lymphocytes. (A) DCs (1×10^5^ cells) were incubated with different concentrations of UV-MV. After 24 hours, the cells were stained for CD150, HLA-DR, CD86 and DC-SIGN using specific antibodies and analyzed by flow cytometry. (B,C) DCs or autologous B cells (BLCL) (5×10^4^ cells) were pre-incubated with mannan and incubated with UV-MV or a positive control peptide overnight. The MV-specific CD4^+^ T-cell clones, GRIM99, GRIM61 and GRIM76 and the non-specific T-cell clone LB5 were added and T cell activation was determined by ELISPOT. GRIM99, GRIM61 and LB5 were dependent on HLA-DQw1 as expressed by the DCs, whereas GRIM76 was HLA-mismatched to the DCs used. Images of the ELISPOT (left) and the mean of the spot counts per well are depicted (right). Error bars represent the standard deviation of duplicates. (D) DCs (1×10^4^ cells) were pre-incubated with mannan, anti-DC-SIGN or anti-CD150 and subsequently incubated with different concentrations of UV-MV. After 6 hours the MV specific CD4^+^ T-cell clone (5×10^4^ cells) was added to the wells. After 24 hours the supernatant was harvested and the amount of IFN-γ analyzed by ELISA. Standard deviations represent the standard deviation of duplicates. A representative experiment out of two is shown.

DC-SIGN is an attachment receptor for MV and mediates infection of DCs through CD150 *in cis* [11]. We investigated whether MV capture by DC-SIGN^+^ DCs also leads to antigen processing and presentation to MV-specific CD4^+^ T-lymphocytes. We performed an antigen presentation assay using MV-specific CD4^+^ T cell clones. [18],[19]. As APCs we used moDCs, expressing high levels of DC-SIGN [20] or an autologous Epstein-Barr virus-transformed B-lymphoblastic cell line (BLCL). The APCs were incubated with different dilutions of MV that was UV-inactivated to exclude DC and T-lymphocyte infection. Subsequently the APCs were co-cultured with the MV-specific CD4^+^ T-cell clones. At the highest concentrations, UV-MV induced DC maturation, since CD86 was upregulated, whereas DC-SIGN was down-regulated (Figure 2A). Notably, in contrast to LPS stimulation, HLA-DR was not upregulated by MV. T-cell activation was measured by the detection of IFN-γ production by ELISPOT and ELISA. We used two different MV-specific T cell clones (GRIM99 and GRIM61) that matched and one that mismatched (GRIM 76) the HLA type of the donor DCs. DCs incubated with UV-inactivated MV specifically activated the HLA-matched MV-specific T cell clones, whereas the mismatched MV-specific T cell clone was not activated (Figure 2B and C). Moreover, an irrelevant T cell clone (LB5) was not activated by the DCs (Figure 2B). Thus, MV capture by DCs leads to specific antigen processing and presentation of MV peptides in the context of MHC-class II molecules. MV-derived antigen presentation by DCs was more efficient at low antigen concentrations than presentation by autologous BLCL (Figure 2B); while the peptide control response was not significantly different (239+/−16 versus 264+/−18).

To investigate whether DC-SIGN and CD150 are involved in antigen presentation, moDCs were pre-treated with specific blocking antibodies to CD150, DC-SIGN and with the C-type lectin inhibitor mannan. Both mannan and anti-DC-SIGN inhibited activation of the MV-specific T-cell clone, demonstrating that DC-SIGN supports MV antigen uptake and processing for antigen presentation (Figure 2B and C). Notably, antibodies against CD150 also inhibited T-cell clone activation, although to a lesser extent than antibodies against DC-SIGN. The role of DC-SIGN and CD150 was not dependent on post-entry effects, since addition of the antibodies just before the T-lymphocytes were added did not affect MV antigen presentation to the CD4^+^ T-cell clone (data not shown). Thus, viral uptake by DC-SIGN and to a lesser extent CD150 leads to virus degradation and antigen presentation of MV-derived antigens to CD4^+^ T-lymphocytes.

### Measles virus replicates in clusters of dendritic cells and T-lymphocytes that are interconnected by infected dendrites

**Figure 3:**
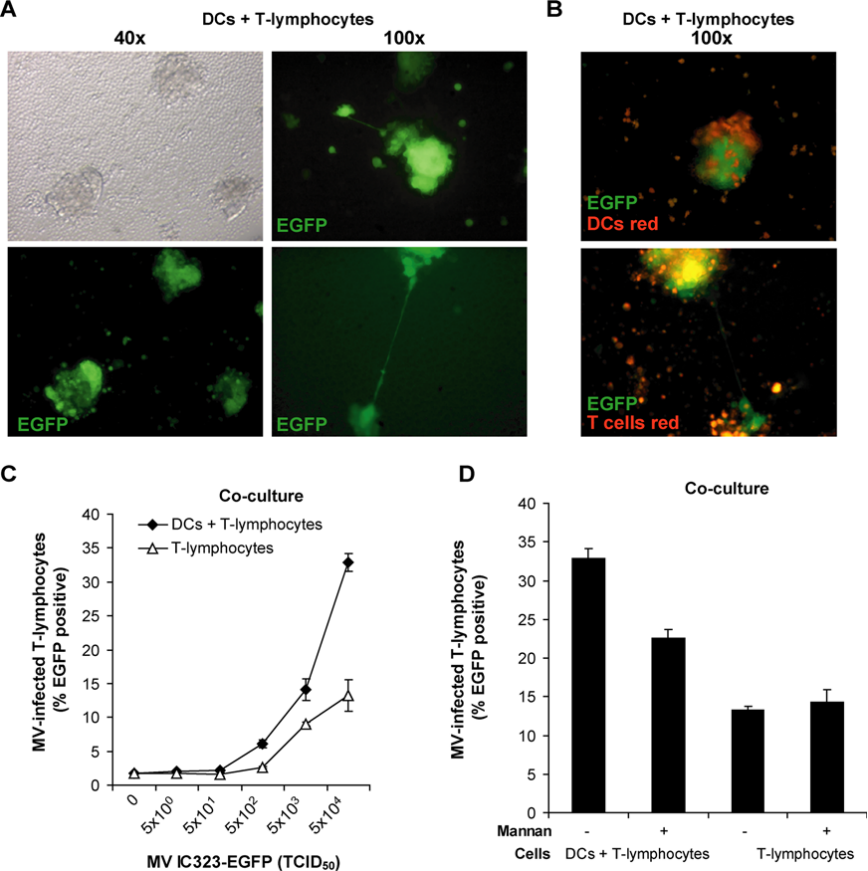
Dendritic cells facilitate measles virus infection of T-lymphocytes in clusters that are interconnected. (A–D) T-lymphocytes (2×10^5^ cells), alone or in the presence of DCs (5×10^4^ cells), were pre-incubated with mannan and infected with MV-IC323-EGFP (TCID_50_ 5×10^4^ unless depicted otherwise) and cultured for 72 hours. (A,B) The infection was analyzed by fluorescence microscopy. The magnification is depicted. (B) DCs or T-lymphocytes were labeled red using the membrane dye PKH26 prior to the experiment and infection by MV-IC323-EGFP is shown by EGFP expression (green). (C,D) The infection of DCs and T-lymphocytes was analyzed by measuring EGFP expression by flow cytometry. Standard deviations represent the standard deviation of triplicates. A representative experiment out of two is shown.

DCs capture HIV-1 via DC-SIGN [9], and facilitate the infection of T-lymphocytes by transferring the virus through the infectious synapse [21]. Since DC-SIGN^+^ DCs and CD150^+^ lymphocytes closely interact in the lymphoid tissues and the upper respiratory tract (Figure 1), we investigated the role of DCs in MV transmission in DC-T-lymphocyte co-cultures. To analyse viral transmission of DCs to T-lymphocytes we used the recombinant MV-IC323-EGFP strain. This MV strain has similar characteristics as its parental IC-B wild-type strain [22], but infected cells produce high amounts of EGFP. The concentration of EGFP in the cells is directly related to the level of virus replication. The entry receptor for this virus is CD150, and not CD46, similar as MV wild-type strains [6]. DCs were infected with MV-IC323-EGFP, and subsequently co-cultured with PHA-stimulated T-lymphocytes expressing high levels of CD150. After two days the infected cells were analyzed by fluorescence microscopy. MV infection induced the formation of large clusters, which contained multiple EGFP^+^ syncytia (Figure 3A). Most infected cells were observed in clusters, and notably long EGFP^+^ dendritic processes were observed that interconnected these clusters (Figure 3A). To investigate which cells were present in the clusters, either DCs or T-lymphocytes were stained with a red dye before infection. Staining of either cell type demonstrated that both infected DCs and T-lymphocytes were present in the EGFP^+^ clusters (Figure 3B), reminiscent of the *in vivo* infection of DCs and T-lymphocytes observed in lymphoid tissues of macaques [2].

To investigate whether DCs enhance the infection of lymphocytes, T-lymphocytes or DC-T-lymphocyte co-cultures were infected with different concentrations of MV-IC323-EGFP and subsequently analyzed by flow cytometry. MV could readily infect activated T-lymphocytes, but addition of DCs enhanced infection two-fold (Figure 3C and D). To investigate the role of DC-SIGN in MV transmission in a DC-T-lymphocyte co-culture, DCs were pre-incubated with mannan and infection was measured. Mannan partially prevented the increased infection of the lymphocytes in the DC-T lymphocyte co-cultures, demonstrating that DC-SIGN is involved in the enhanced infection in the DC-T lymphocyte co-culture, probably by increasing viral transmission to T-lymphocytes (Figure 3D).

### Dendritic cells transmit measles virus to T-lymphocytes independently of *de novo* virus production

**Figure 4:**
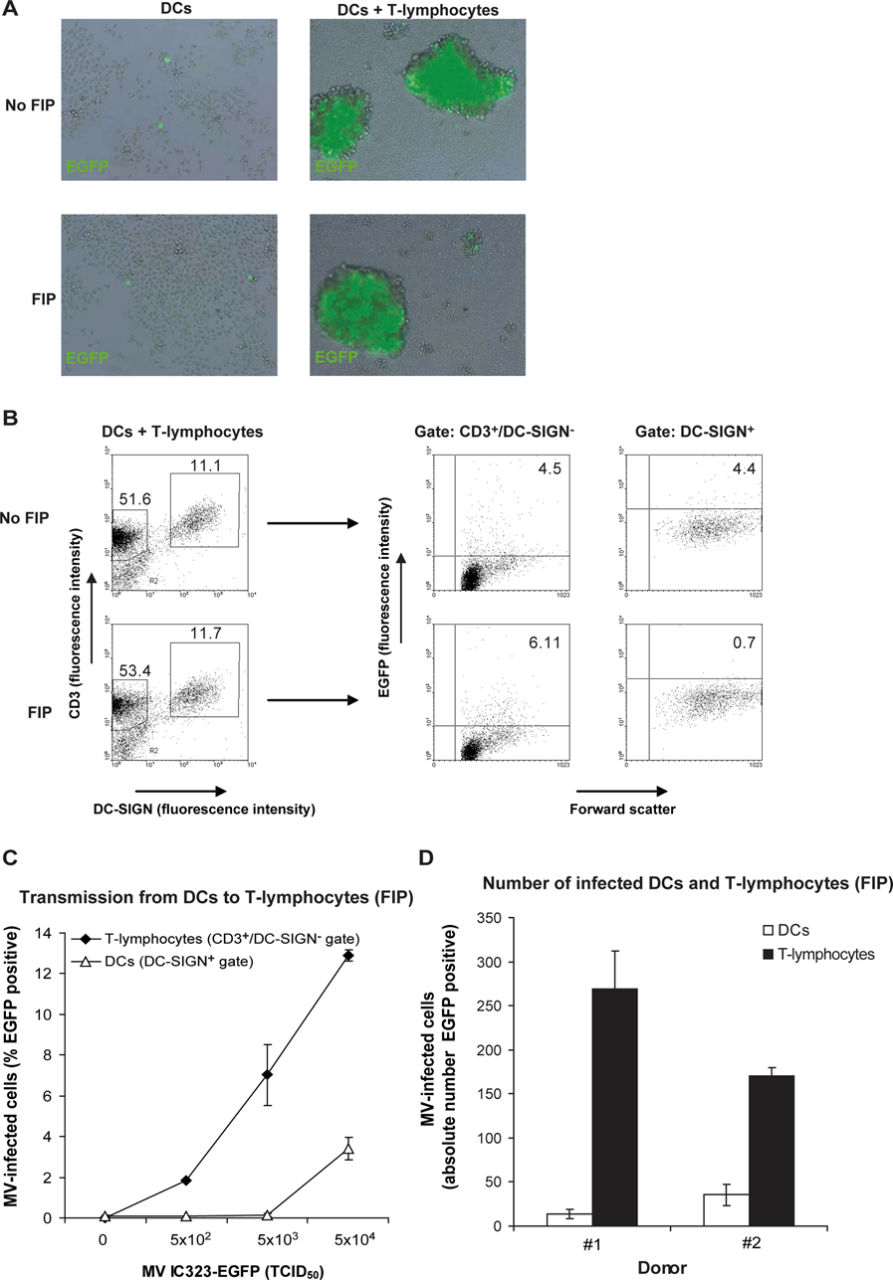
Dendritic cells mediate viral transmission of measles virus independently of *de novo* virus production. (A–D) DCs (5×10^4^ cells) were incubated with MV-IC323-EGFP (5×10^4^TCID_50_, unless depicted otherwise) and after 2 hours the cells were extensively washed. T-lymphocytes (2×10^5^ cells) were added and if indicated the fusion inhibitory peptide (FIP) was added after 2 hours. After 72 hours the infection was analyzed by (A) fluorescence microscopy and (B–D) flow cytometry. (A) The cultures are depicted as an overlay of EGFP and brightfield. (B,C) The cells were harvested, washed and stained for CD3 and DC-SIGN. EGFP expression was measured by flow cytometry. (B) The percentage gated is depicted in the regions or quadrants. (C) Transmission of different concentrations of MV IC323-EGFP is analyzed. (D) The absolute numbers of EGFP^+^ DCs and T-lymphocytes in the analyzed samples were calculated and depicted. Error bars represent the standard deviation of triplicates. A representative donor out of seven is shown.

To investigate whether DCs transmit MV to their target cells, DCs were incubated with MV-IC323-EGFP for two hours, washed extensively to remove unbound virus and subsequently co-cultured with activated T-lymphocytes. In DC cultures without T-lymphocytes, low percentages of MV-infected DCs were detected, whereas in the DC-T lymphocyte co-cultures large clusters of MV-infected cells and syncytia were observed (Figure 4A upper panels). These data strongly suggest that DCs capture MV and transmit the virus to T-lymphocytes independently of *de novo* synthesis of virus by infected DCs, since only a few infected DCs were observed (Figure 4A). This process is referred to as *trans-*infection. However, HIV-1 studies have shown that DCs can also mediate transmission of *de novo* synthesized HIV-1 [10]. DCs in the respiratory tract *in situ* express high levels of DC-SIGN and no or low amounts of CD150 (Figure 1), suggesting that DCs are not productively infected by MV. Therefore, we investigated whether DCs mediate *trans-*infection. As demonstrated, DCs transmit MV efficiently to T-lymphocytes in a co-culture (Figure 4A) but *de novo* synthesis in DCs cannot be excluded, since both DCs and T-lymphocytes are infected in the DC-T lymphocyte co-culture (Figure 4B). To exclude *de novo* synthesis of virus in DCs, we used the fusion inhibitor peptide (FIP, 200µM) [23], which was added to the co-cultures 2 hours after addition of the T-lymphocytes to MV-infected DCs. We observed large clusters of EGFP^+^ cells in the presence of FIP (Figure 4A). FIP prevents fusion of MV with cell membranes and of membranes of MV-infected cells with those of neighbouring cells. Therefore FIP blocks infection and syncytium formation [24]. Thus, T-lymphocytes expressing EGFP must have been infected during the 2 hours co-cultivation with MV-infected DCs before FIP was added (Figure 4B). This is a time frame that excludes *de novo* synthesis of MV by the DCs. In contrast to the condition without FIP, no syncytium formation was observed in DCs and T-lymphocytes cultured in the presence of FIP, confirming that FIP indeed prevented fusion. Moreover, incubation of DCs with different concentrations of MV demonstrated that T-lymphocytes were the major MV-infected cell population in the DC-T lymphocyte co-culture (Figure 4C), demonstrating that DCs mediate *trans-*infection. To determine the efficiency of the *trans*-infection, the absolute number of infected cells was calculated (Figure 4D). A 6-fold higher number of T-lymphocytes compared to DCs were infected in the co-cultures. This demonstrates that *trans-*infection of T-lymphocytes by DC-bound MV is more efficient than *cis-*infection of DCs. Thus, DCs efficiently mediate transmission of MV to T-lymphocytes, and this process primarily occurs independently of *de novo* synthesis of MV.

### Measles virus *trans-*infection of T-lymphocytes by dendritic cells is dependent on DC-SIGN, but not CD150

**Figure 5:**
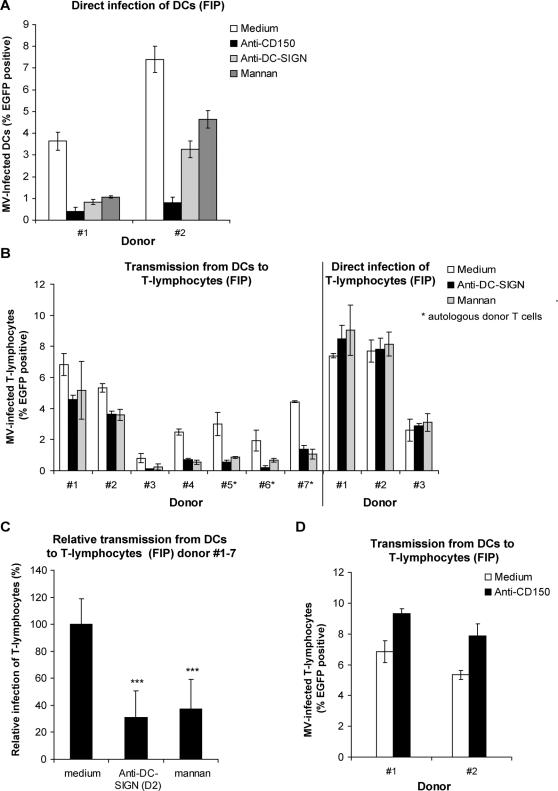
*Trans-*infection of measles virus by dendritic cells is dependent on DC-SIGN, but not on CD150. (A–D) DCs (5×10^4^ cells) or (B) T-lymphocytes from different donors were pre-incubated with mannan, anti-DC-SIGN or anti-CD150 (for donor #1 and #2), before incubation with MV-IC323-EGFP (5×10^4^ TCID_50_). (A) The cells were cultured in the presence of FIP for 3 days and analyzed by flow cytometry. (B–D) After 2 hours the cells were washed and T-lymphocytes (2×10^5^ cells) were added. For donor #5–7 autologous T-lymphocytes were used as target cells. The fusion inhibitory peptide (FIP) was added after 2 hours. After 72 hours the cultures were analyzed by flow cytometry. (B) Transmission by different donors of DCs is depicted. As a control for the specificity of mannan and anti-DC-SIGN T-lymphocytes were pre-treated and directly infected. Error bars represent the standard deviation of triplicates. (C) The results of the different donors in (B) (#1–7) were normalized to the medium control to determine the statistical differences by ANOVA. Error bars represent the standard deviation of the mean of the different donors, *** = p<0,01 versus the medium control.

DC-SIGN and CD150 are both important for binding of MV to DCs and subsequent infection. Indeed, MV-infection of DCs is inhibited by antibodies against CD150 and DC-SIGN, as well as by mannan (Figure 5A) [11]. MV transmission by *de novo* synthesis of MV particles depends on infection of DCs and therefore these data suggest that transmission through *de novo* synthesis of virus is dependent on both CD150 and DC-SIGN.

To investigate whether DC-SIGN is involved in MV *trans-*infection of T-lymphocytes, DCs were pre-treated with mannan or antibodies against DC-SIGN and transmission was measured in the presence of FIP. In all donors, both mannan and antibodies against DC-SIGN inhibited *trans-*infection (Figure 5B), although donor variations were observed. Direct infection of T-lymphocytes after pre-treatment with the blocking agents demonstrated that these compounds do not interfere with the infection of the target cells (Figure 5B). Allogeneic DCs induce T-lymphocyte activation and as such might increase CD150 expression and subsequently MV infection of T-lymphocytes. Therefore, we used autologous T-lymphocytes for donors #5–7. In this setting, *trans-*infection was also observed and was dependent on DC-SIGN (Figure 5B), indicating that *trans*-infection is independent of T-lymphocyte activation. To analyze whether these differences were significantly different throughout the donors, data were normalized to the medium condition (Figure 5C), demonstrating that DC-SIGN is important for transmission of MV from DCs to T-lymphocytes (Figure 5C).

Since CD150 is important for MV binding to DCs and subsequent infection, we investigated whether this receptor is also important for *trans-*infection of T-lymphocytes. Strikingly, antibodies against CD150 did not block the *trans-*infection and even slightly increased the transfer to T-lymphocytes (Figure 5D). These results demonstrate that DC-SIGN mediates both DC infection *in cis* and *trans-*infection, whereas CD150 is only involved in infection, and thus CD150 and DC-SIGN have distinct roles in MV transmission by DCs to T-lymphocytes.

### DCs transmit MV to both CD4^+^ and CD8^+^ T-lymphocytes

**Figure 6:**
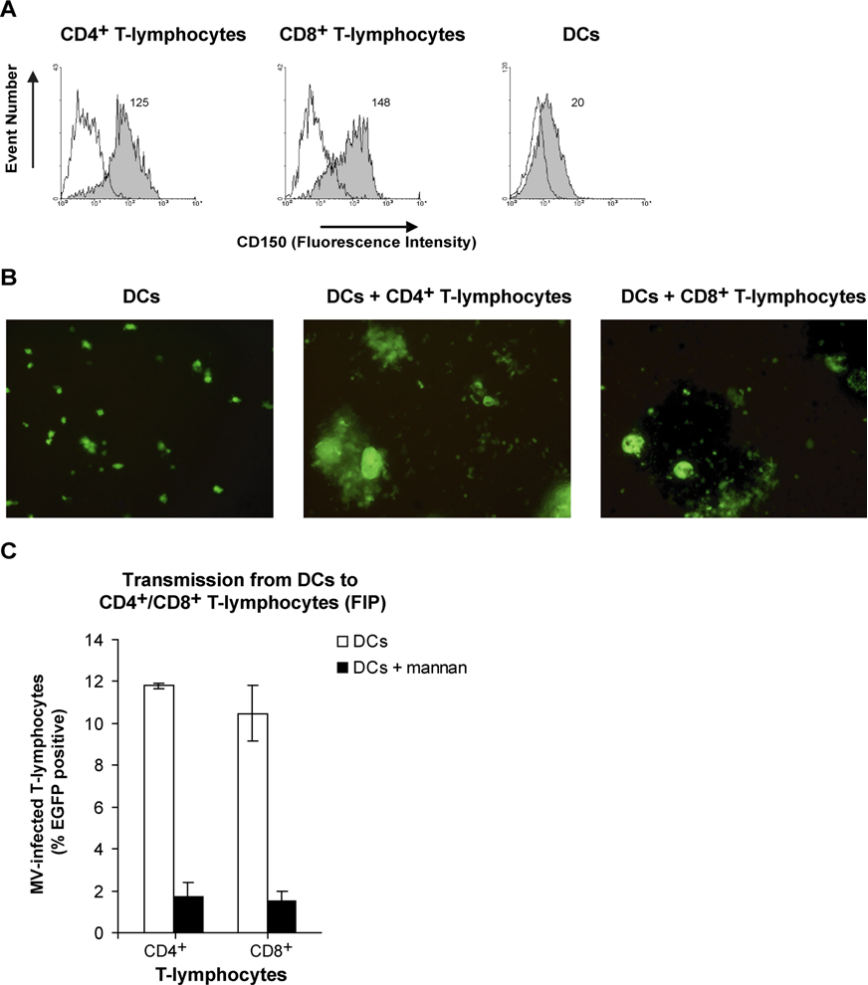
Dendritic cells mediate transmission of measles virus to both CD4^+^ and CD8^+^ T-lymphocytes. (A) PBMCs were activated for three days, and the cells were enriched for CD4^+^ T-lymphocytes or CD8^+^ T-lymphocytes. T-lymphocytes and DCs are stained for the expression of CD150 and analyzed by flow cytometry. Open histograms represent isotype-control and filled histogram the specific antibody staining. The mean of the specific staining is depicted. (B,C) DCs (5×10^4^ cells) were pre-incubated with mannan before incubation with MV-IC323-EGFP (5×10^4^ TCID_50_). After 2 hours the cells were washed and cultured in the presence of the CD4^+^ or CD8^+^ T-lymphocytes for three days. (B) The cultures were analyzed by fluorescence microscopy and pictures were made at a magnification of 200× (C) The fusion inhibitory peptide (FIP) was added after 2 hours. The cells were harvested and EGFP expression was measured by flow cytometry. Standard deviations represent the standard deviation of triplicates. A representative experiment out of two is shown.

In peripheral blood of experimentally infected macaques, MV infection of both CD4^+^ and CD8^+^ T-lymphocytes was observed [2]. Therefore we investigated whether DCs mediate transmission of MV to both T-lymphocyte subsets *in vitro*. CD4^+^ and CD8^+^ T-lymphocytes were purified from PHA-stimulated human peripheral blood mononuclear cells (PBMCs), and both expressed high levels of CD150 (Figure 6A). DCs were infected with MV-IC323-EGFP, and after extensive washing, co-cultured with either CD4^+^ or CD8^+^ T-lymphocytes. In both co-cultures, EGFP expression was observed in large clusters containing EGFP^+^ syncytia, demonstrating that DCs mediate transmission to both T-lymphocyte subsets (Figure 6B). To measure whether DCs mediate *trans-*infection, FIP was added two hours after addition of the T-lymphocytes to the MV-infected DCs. *Trans-*infection to both subsets is efficient and mediated by DC-SIGN, since pre-treatment of DCs with mannan inhibited infection of T-lymphocytes in both cultures (Figure 6C). Thus, DCs transmit MV to both CD4^+^ and CD8^+^ T-lymphocytes.

## Discussion

Two hallmarks of measles are that the virus is highly contagious and infection results in strong MV-specific cellular immune responses. Both viral transmission and antigen presentation should therefore occur through efficient and robust processes. Since DCs are professional APCs and have been demonstrated to mediate transmission of several viruses, we have investigated the role of DCs in both processes for measles. Here we have shown that a subset of DCs, the DC-SIGN^+^ DCs, mediates both transmission and antigen presentation of MV to T-lymphocytes. The receptors DC-SIGN and CD150 are both involved in DC infection and antigen presentation, whereas only DC-SIGN is involved in MV *trans-*infection of T-lymphocytes.

DC-SIGN^+^ DCs have previously been identified in several sub-epithelial and lymphoid tissues [9],[13]. Here, we have identified DC-SIGN^+^ DCs in the sub-epithelial tissues of the human mouth, pharynx, trachea and bronchi, and the presence of scattered DC-SIGN^+^ cells in the lung. CD150 is expressed on *in vitro* cultured macrophages and DCs and is increased upon maturation [16],[17],[25]. However, CD150 was previously not detected on immature DCs in skin and lung *in situ* [26] and we could not detect CD150 on DC-SIGN^+^ DCs in respiratory epithelia, suggesting that these DCs are not susceptible to MV infection and DCs capture MV through DC-SIGN. However, expression of low levels of CD150 that might support MV infection cannot be excluded using immunofluorescence microscopy. DC-SIGN strongly enhances infection of DCs through CD150 [11], and therefore low levels of CD150 might be enough for efficient infection of DCs.

Different mechanisms are involved in virus transmission by DC-SIGN, since HIV-1 capture by DC-SIGN can result both in *cis* and *trans-*infection [9],[27]. However, DC-SIGN binding of HIV-1 also leads to virus degradation and presentation in the context of MHC [28],[29]. Interestingly, we observed that both DC-SIGN and CD150 are involved in MV processing and presentation of MV-derived peptides to the MV-specific CD4^+^ T-cell clone GRIM99. B cells are able to present MV antigens to T cells [30]. BLCL express high levels of CD150, but no DC-SIGN and MV capture leads to virus degradation and presentation of MV peptides to the autologous CD4^+^ T-cell clone and this was previously demonstrated to be dependent on endocytosis [30]. Notably, DCs were more efficient in antigen processing and presentation than the autologous BLCL and antibodies against DC-SIGN inhibited antigen presentation to a larger extent than antibodies against CD150. Thus, although both DC-SIGN and CD150 are involved, DC-SIGN is more important for antigen presentation of MV by DCs.

MV is a highly contagious virus [1] suggesting that it has a very efficient entry into the respiratory tissues. However, the number of CD150^+^ target cells in the respiratory tract is low, suggesting that these are not the first targets for MV at the site of entry. In contrast, the tissue is lined with DC-SIGN^+^ DCs and these DCs are better candidates since DC-SIGN^+^ DCs efficiently capture MV and mediate transmission of MV to T-lymphocytes *in vitro*. MV transmission to non-stimulated lymphocytes by DCs was inefficient (data not shown), which is in line with a previous report that demonstrated that transmission of MV from DCs to T cells isolated from blood is inefficient [31]. This is probably due to low expression of CD150 on blood-derived lymphocytes. However in lymphoid tissues, where DCs migrate to, CD150 is highly expressed on T and B-lymphocytes and on monocytes [7],[32].

Recently it was demonstrated that DCs specifically transmit HIV-1 to HIV-1-specific T-lymphocytes [29] suggesting that immunological synapse formation enhances viral transmission, due to prolonged interactions during antigen presentation and T cell activation. However, we did not observe differences between transmission to autologous- and allogeneic T-lymphocytes, suggesting that prolonged immunological synapse interactions occurring during allogeneic but not autologous T-DC interactions are not necessary for MV transmission. This might be due to differences in infectivity between both viruses.

MV transmission can occur independently of *de novo* synthesis of virus in DCs (referred to as *trans-*infection). Using specific blocking antibodies, we have demonstrated that *trans-*infection is dependent on DC-SIGN but not on CD150. This is physiologically relevant, since DC-SIGN^+^ DCs in the respiratory tract express no or low levels of CD150, and are therefore not susceptible to MV infection. Both DC-SIGN and CD150 are important for binding of MV to DCs [11]. However, our data show that binding of MV to DC-SIGN and CD150 results in different internalization pathways. Although both DC-SIGN and CD150 lead to virus degradation for antigen presentation, CD150 binding also results in viral entry, whereas only the interaction of DC-SIGN with MV leads to viral protection for *trans-*infection. Indeed, inhibition of CD150 resulted in enhanced *trans*-infection due to less degradation or fusion and therefore increasing the amount of virus for the DC-SIGN-mediated transmission. Several donors were tested for the involvement of DC-SIGN in *trans-*infection of MV. The contribution of DC-SIGN varied between the donors, suggesting that another attachment receptor might play a role, such as syndecan-3 for HIV-1 [33]. In macaques, both CD4^+^ and CD8^+^ T-lymphocytes are infected during the viraemic phase of measles disease [2]. It is unclear whether viral transmission can also occur to CD8^+^ T lymphocytes. Interestingly, we observed MV transmission by DCs to both CD4^+^ and CD8^+^ T-lymphocytes, indicating the formation of an infectious synapse between DCs and CD8^+^ T-lymphocytes, similar to DCs and CD4^+^ T-lymphocytes [21].

During MV infection in macaques, lymphoid tissues are major sites of MV replication [2]. In human lymphoid tissues, CD150^+^- and DC-SIGN^+^ cells are in close contact, which can contribute to massive replication of MV. This is supported by our *in vitro* observations that DC-SIGN^+^ DCs enhance infection of T-lymphocytes in co-cultures and MV infection is predominantly observed in the clusters of DCs and T-lymphocytes. Notably, long EGFP^+^ dendrites were frequently observed between clusters, suggesting that viruses spread between clusters through these dendrites. Although these dendrites might be a MV-specific effect, which facilitates virus spread, these dendritic processes might also have a physiological function in the immune system, such as the interchange of antigenic information. The fact that MV infection in DC-T lymphocyte co-cultures was observed in clusters was highly reminiscent to the infectious foci that we have previously observed in tissues of infected macaques [2]. This pattern and enhanced infection in DC-T lymphocyte co-cultures suggest that the virus is much more efficiently transmitted by direct cell-cell contact than as cell-free virus.

In contrast to the APCs in the peripheral tissues, the borders of the medullary sinuses of the lymph node contain a population of cells that express high amounts of both DC-SIGN and CD150. These DC-SIGN^+^ cells have been previously shown to express CD68 and lack the expression of DEC205, suggesting that these cells are medullary macrophages [13],[34]. These cells are in contact with the lymph and therefore encounter tissue-derived antigens. Based on the high expression of both CD150 and DC-SIGN, and the fact that DC-SIGN can enhance infection *in cis* [11], it is likely that these cells become infected during measles and might contribute to further virus transmission.

In conclusion, these data provide us with an alternative view on how MV might disseminate from the site of infection to their main target cells, the lymphocytes: DC-SIGN^+^ DCs, which are abundantly present in the sub-epithelial tissues of the respiratory tract, capture MV and process the virus for antigen presentation, but a part of the virus escapes from degradation. In previous studies dendrites of sub-epithelial DCs have been shown to pass the tight junctions of the epithelium of the gut and respiratory tract and sample the mucosal surfaces [35], which could result in efficient capture of the virus. Moreover, MV induces activation of the DC via TLRs [36], which will induce migration of DCs from the peripheral tissues to the lymphoid tissues. Although DCs might encounter CD150^+^ cells in the underlying mucosal tissues, the abundant expression of CD150 in lymphoid tissues strongly suggests lymphoid tissues as the major site of MV transmission and replication. A similar mechanism might play a role in spreading the virus throughout the body, even to privileged tissues such as the brain, and could therefore be involved in complications such as subacute sclerosing panencephalitis (SSPE). Moreover, during viraemia, DCs might increase infection and tissue destruction. In the future, *in vivo* studies will be required to prove the importance of MV transmission by DCs.

## Materials and Methods

### Antibodies

The following antibodies were used: CD150-specific mouse antibody 5C6 [37], DC-SIGN-specific mouse antibodies AZN-D1 and AZN-D2 [9], DCN46 and DCN46 conjugated with PE (BD Pharmingen, San Diego, CA, USA), CD3-specific mouse antibody SK-7 conjugated with PerCP (BD Pharmingen, San Diego, CA, USA), goat anti-mouse IgG antibody conjugated with PO (Jackson Immunoresearch, West Grove, PA, USA), HLA-DR- (Immu357) and CD86- (HA5.2B7) specific mouse antibodies conjugated with PE (Immunotech, Marseille, France), goat anti-mouse antibody conjugated with FITC (Zymed Laboratories Inc., South San Fransisco, CA. USA), Alexa488- or Alexa594-labeled anti-mouse antibodies (Molecular probes, Eugene, OR, USA).

### Cells

Vero-CD150 cells [38] were grown in Dulbecco's Modified Eagle's Medium (DMEM; Gibco Invitrogen, Carlsbad, CA, USA) supplemented with 4500 mg/L glucose; 110 mg/l sodium pyruvate; 4 mM L-glutamine; 10% heat-inactivated fetal calf serum (FCS); 20 U/ml penicillin and 20 µg/ml streptomycin, at 37°C with 5% CO_2_. The CD4^+^ HLA-DQw1-restricted T-cell clone GRIM99 [18],[19] recognizes an epitope in the MV fusion protein (EVNGVTIQV). GRIM61 and GRIM99 are also MV-specific CD4+ T cell clones, for which the epitopes have not been mapped (Van Binnendijk JI 1989). LB5 is a CD4+ T cell clone of unknown specificity (unpublished). All clones were cultured in RPMI-1640 (RPMI 1640, Gibco Invitrogen, Carlsbad, CA, USA) supplemented with 4 mM L-glutamine; 10% heat-inactivated human AB serum (Sigma-Aldrich, St. Louis, MO, USA); 20 U/ml penicillin, 20 µg/ml streptomycin and 10^−5^ M 2-mercapto-ethanol in 96-well round bottom plates. Epstein-Barr virus-transformed B-lymphoblastic cell line (BLCL-GR) [18],[19] was used as autologous APC, and was cultured in RPMI 1640 supplemented with L-glutamine, penicillin, streptomycin and 10% FCS. Immature moDCs were cultured as described before [39]. In short, human blood monocytes were isolated from buffy coats by Ficoll density centrifugation, followed by selection of CD14^+^ cells using magnetic beads (MACS, Milteny Biotec GmbH, Bergisch Gladbach, Germany). Purified monocytes were cultured in RPMI-1640 medium supplemented with 4 mM L-glutamine; 10% FCS; 20 U/ml penicillin and 20 µg/ml streptomycin and differentiated into immature moDCs in the presence of IL-4 and GM-CSF (500 and 800 U/ml, respectively; Schering-Plough, Brussels, Belgium).

PBMCs were isolated from buffy coats by Ficoll density centrifugation, activated with phytohemagglutinin (3 µg/ml; Sigma-Aldrich, St. Louis, MO, USA), and cultured in complete RPMI-1640 medium. At day 3 the cells were washed and cultured with IL-2 (100 units/ml). As determined by flow cytometry >80% of the activated PBMC were CD3^+^ and therefore these cells are referred to as T-lymphocytes throughout the text. The CD4^+^ and CD8^+^ T-lymphocytes were enriched at day 3 after PHA stimulation by negative selection using MACS beads. To label DCs and T-lymphocytes, the cells were stained with the PKH26 red fluorescent cell linker kit (Sigma-Aldrich, St. Louis, MO, USA) for general cell membrane labelling, according to the manufacturers protocol.

### Viruses

The recombinant MV strain IC323-EGFP [40] was propagated on Vero-CD150 cells. For virus production, Vero-CD150 cells were infected with a multiplicity of infection (MOI) of 0.01 in DMEM supplemented with 2% of FCS. After 90 minutes cells were washed to remove unbound virus and were subsequently grown in DMEM supplemented with 10% FCS. Cells and supernatant were harvested when 80% cytopathic effect was observed. To release cell-bound virus, the cells were sonicated (3 times, 10 seconds, Sonicor Instrument Corporation, Copiaque, N.Y., USA). The cells were centrifuged (10 minutes, 1000 g) and the supernatant was snap-frozen in liquid nitrogen before titration on Vero-CD150 cells. The titer of the virus-stock used was 1×10^6^ TCID_50_/ml. Purified MV Edmonston with a concentration of approximately 1 mg/ml, was inactivated by UV-irradiation (30 minutes, 15W 312 nm) and is referred to as UV-MV throughout the text.

### Immunofluorescence Microscopy

Tissues of healthy human donors were obtained through the pathology department of the VU University Medical Center, according to the institutional ethical guidelines. Cryosections (7 µm) were fixed with 100% acetone and stained with primary antibody combinations against DC-SIGN (DCN46, IgG2b, 10 µg/ml) and anti-CD150 (5C6, IgG1, 10 µg/ml) or a buffer control for 18 hours at 4°C. Sections were counterstained with isotype-specific Alexa488- or Alexa594-labeled anti-mouse antibodies. Nuclei were stained with Hoechst (Molecular Probes, Eugene, OR, USA). After mounting, sections were examined with a Nikon Eclipse E800 fluorescence microscope and recordings were made with a digital NIKON DXM1200 camera. Two persons used the photographs to quantify the staining in the different tissues independently. To determine the density of DC-SIGN^+^- or CD150^+^ cells, the number of positive cells was divided by the total number of cells, based on the nuclei staining. To determine the co-localization, the double-stained cells were divided by the total number of stained cells. Based on the control sections, autofluorescence was often observed in the lower respiratory tract and is indicated in the pictures.

### Antigen Presentation Assays

Monocytes were isolated from an HLA-DQw1-matched donor using CD14 MACS beads and differentiated into immature DCs as described above. Subsequently, these DCs or autologous BLCL-GR (5×10^3^ cells) were used as APCs in an interferon-γ (IFN-γ) ELISPOT assay as previously described [41]. Briefly, APCs were plated into 96-well v-bottom plates in complete RPMI-1640 containing IL-4 and GM-CSF and pre-incubated with mannan (0,25 mg/ml) for 30 minutes at 37°C. Next, the cells were incubated with different dilutions of UV-MV at 37°C or a positive control peptide (EVNGVTIQV; 1 µM). After overnight incubation the CD4^+^ T-cell clones were added to the APCs (5×10^3^ cells per well), the plates were briefly centrifuged (1 minute, 300 g) and subsequently incubated at 37°C for 1.5 hour. Subsequently the cells were transferred to nylon bottom plates (Nalge Nunc International, Rochester, NY) coated with a monoclonal antibody specific for human IFN-γ (1-D1K; Mabtech, Stockholm, Sweden), and incubated at 37°C for five hours. Finally, plates were washed with phosphate-buffered saline (PBS) containing 0.05% Tween 20 (Merck, Darmstadt, Germany). Spots were visualized by incubation with a secondary biotinylated mAb against IFN-γ (7-B6-1; Mabtech), followed by staining with streptavidin–alkaline phosphatase (Mabtech), and nitroblue tetrazolium–5-bromo-4-chloro-3-indolyl-phosphate (Kirkegaard & Perry Laboratories, Gaithersburg, MA, USA). Finally, the color reaction was stopped by washing the plates with water and spots were counted with an automated ELISPOT reader (automated ELISAspot assay video analysis systems; distributed by Sanquin Reagents, Amsterdam, The Netherlands).

In parallel, the same APCs were also used to stimulate the same T-cell clone for IFN-γ production in supernatant. Briefly, APCs (1×10^4^ cells) were used to stimulate the T-cell clone (3×10^4^ cells) in round-bottom plates, and were incubated at 37°C for 24 hours before supernatants were harvested. To determine the contribution of DC-SIGN and CD150, the APCs were pre-incubated with mannan (0,25 mg/ml), anti-DC-SIGN (AZN-D2; 20 µg /ml) or anti-CD150 (5C6; 20 µg/ml) for 30 minutes at 37°C. The IFN-γ concentrations in the supernatants were determined by ELISA (Biosource International, CA, USA).

### MV Infection, Transmission and Co-culture Assays

For infection and transmission assays DCs (5×10^4^ cells) were seeded in a V-bottom plate and pre-incubated with mannan (0,25 mg/ml), anti-DC-SIGN (AZN-D2; 20 µg/ml) or anti-CD150 (5C6; 20 µg/ml) for 30 min. at 37°C, before incubation with MV-IC323-EGFP at 37°C for 2 hours (5×10^4^ TCID_50_, unless stated otherwise). After 2 hours the cells were washed and transferred to a flat-bottom plate. To measure transmission, activated T-lymphocytes (2×10^5^ cells) were added. If indicated the fusion inhibitory peptide (FIP) Z-d-Phe-L-Phe-Gly-OH (Z-FFG; 0.2 mM; Bachem, Heidelberg, Germany) was added 2 hours later. For co-culture assays T-lymphocytes (2×10^5^ cells), either or not together with DCs (5×10^4^ cells), were pre-incubated with 0,25 mg/ml mannan and infected with different concentrations of MV-IC323-EGFP.

All cells were cultured for three days at 37°C in complete RPMI-1640 containing IL-4 and GM-CSF. The cultures were monitored using a Leica DMIL fluorescence microscope, and pictures were taken using a Leica DFC 320 camera (Leica Microsystems, Wetzlar, Germany). At day 3 the cells were harvested, washed and fixed with 4% PFA and EGFP expression was measured by flow cytometry. DCs had higher autofluorescence compared to T-lymphocytes the EGFP^+^ gate for both population was set at the uninfected control sample. To determine the infection of specific cell populations, the cells were stained with directly labeled antibodies against DC-SIGN and CD3 before analysis. The absolute number of infected DCs (DC-SIGN^+^/EGFP^+^) and T cells (CD3^+^/EGFP^+^) of the total counted sample by flow cytometry ( = 10^5^ events) were used to determine the efficiency of transmission.

### Statistical Analysis

To determine the variation in MV transmission among the different DC donors, the infection of T-lymphocytes was normalized to the “medium” control condition, which was set at 100%. Subsequently, the percentages of infection from the different donors were used in a one-way analysis of variance (ANOVA). When the overall *F* test was significant, differences among the donors were further investigated with the post hoc Bonferroni test using Graphpad Prism software. A probability of *p*<0.05 was considered statistically significant.

## Supporting Information

Figure S1 DC-SIGN^+^-dendritic cells are present in the sub-epithelial tissues of the respiratory tract. Cryosections of different tissues from healthy donors were stained for the expression of DC-SIGN (green) and CD150 (red) using specific antibodies, and for the nuclei using Hoechst (blue). The sections were analyzed by fluorescence microscopy. Representative photos with a magnification of 100× are depicted (e = epithelium; s = sub-epithelial tissue, arrow = autofluorescence).
(6.29 MB TIF)
