# Supplementary figures and images for: DC-SIGN and CD150 Have Distinct Roles in Transmission of Measles Virus from Dendritic Cells to T-Lymphocytes
Source: PLoS Pathog. 2008 Apr 18;4(4):e1000049. doi: 10.1371/journal.ppat.1000049 (PMC2277461; doi:10.1371/journal.ppat.1000049)

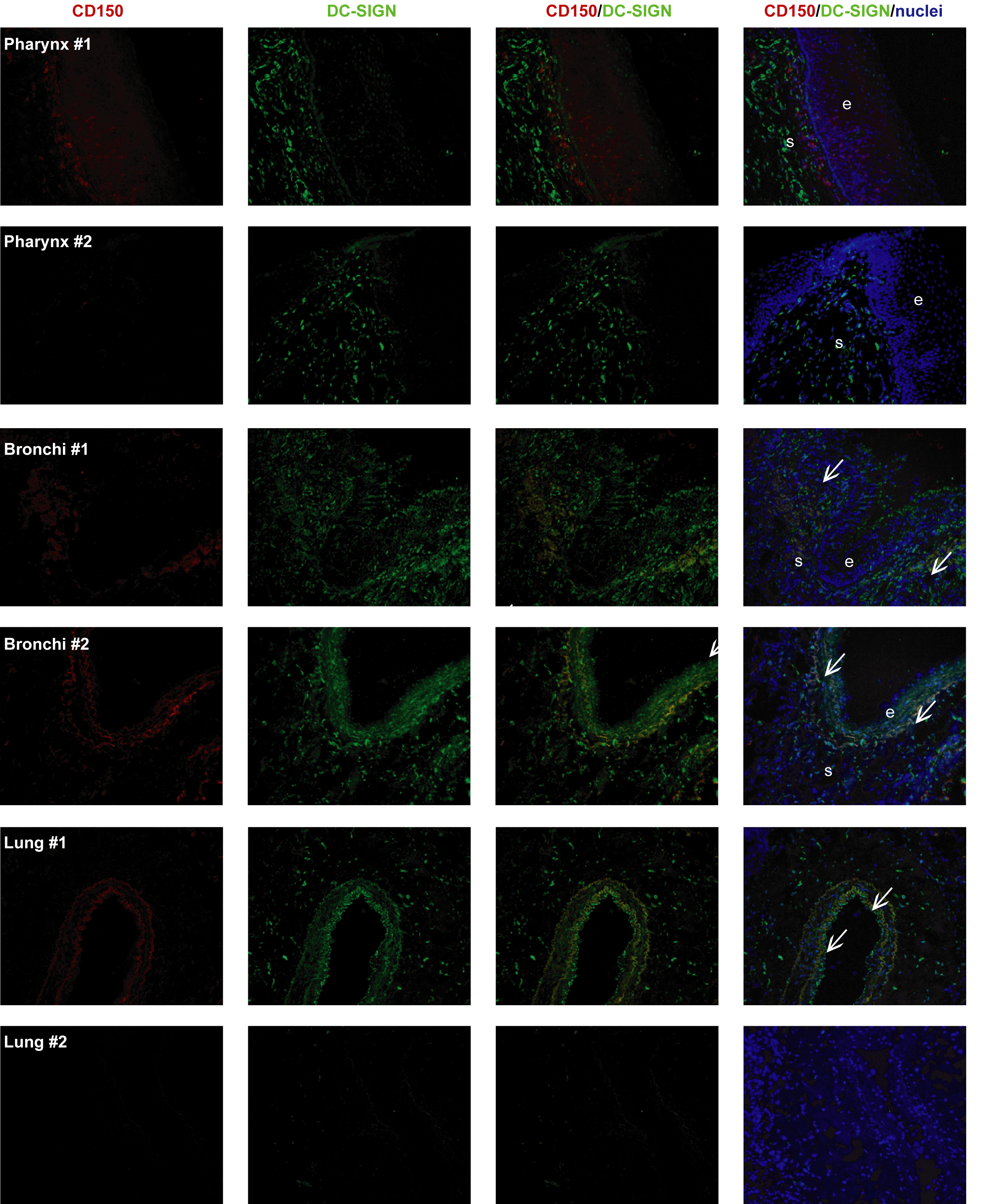

Supplement: Figure S1 — DC-SIGN+-dendritic cells are present in the sub-epithelial tissues of the respiratory tract. Cryosections of different tissues from healthy donors were stained for the expression of DC-SIGN (green) and CD150 (red) using specific antibodies, and for the nuclei using Hoechst (blue). The sections were analyzed by fluorescence microscopy. Representative photos with a magnification of 100× are depicted (e = epithelium; s = sub-epithelial tissue, arrow = autofluorescence). (6.29 MB TIF) [file ppat.1000049.s001.tif]
